# Assessing the relationship between lipoprotein(a) levels and blood pressure among hypertensive patients beyond conventional measures. An observational study

**DOI:** 10.1038/s41598-024-65231-w

**Published:** 2024-06-23

**Authors:** Nestor Vazquez-Agra, Anton Cruces-Sande, Sofia Barbosa-Gouveia, Jose-Enrique Lopez-Paz, Miguel Camafort, Emilio Casariego-Vales, Antonio Pose-Reino, Alvaro Hermida-Ameijeiras

**Affiliations:** 1 Department of Internal Medicine, University Hospital of Santiago de Compostela, A Choupana Street, 15706 Santiago de Compostela, A Coruña Spain; 2 Health Research Institute of Santiago de Compostela (IDIS), A Choupana Street, 15706 Santiago de Compostela, A Coruña Spain; 3 Department of Psychiatry, Radiology, Public Health, Nursing and Medicine, Faculty of Medicine, University of Santiago de Compostela, 15706 Santiago de Compostela, A Coruña Spain; 4 Laboratory of Neurochemistry, Department of Biochemistry and Molecular Biology, Faculty of Medicine, University of Santiago de Compostela, 15706 Santiago de Compostela, A Coruña Spain; 5 Unit of Diagnosis and Treatment of Congenital Metabolic Diseases, Department of Paediatrics, University Hospital of Santiago de Compostela, 15706 Santiago de Compostela, A Coruña Spain; 6 Department of Internal Medicine, Hospital Clinic de Barcelona, 08036 Barcelona, Spain; 7 CIBEROBN, Carlos III Health Institute (ISCIII), 28029 Madrid, Spain

**Keywords:** Blood pressure monitoring, Hypertension, Lipoprotein(a), Cardiology, Endocrinology, Risk factors

## Abstract

High lipoprotein(a) (Lp(a)) levels are associated with an increased risk of arterial hypertension (AHT) and atherosclerotic cardiovascular disease. However, little is known about the detailed profile of AHT based on Lp(a) levels. This observational study focused on elucidating the relationship between Lp(a) concentrations and specific indices obtained from 24-h ambulatory blood pressure (BP) monitoring in hypertensive patients over 18 years of age. We gathered and analyzed data on BP indices along with demographic, epidemiological, clinical, and laboratory variables from 227 hypertensive patients, median age 56 years, including 127 women (56%). After comparing hypertensive patients with Lp(a) levels above and below 125 nmol/L, we found that a 10 mmHg increase in nocturnal systolic BP and all pulse pressure indices (24-h, daytime, and night-time) was associated with an increased risk of high Lp(a) levels by more than 20% and 40%, respectively. Similarly, each 10% increase in the area under the function over time of nocturnal diastolic BP dipping was associated with more than a 30% decrease in the odds of belonging to the elevated Lp(a) levels category. Additionally, Lp(a) levels above 125 nmol/L were associated with higher 24-h, daytime, and night-time systolic BP and pulse pressure load. The relationship between Lp(a) and AHT appears to extend beyond conventional BP measurements, which may be relevant given the prognostic implications of nocturnal BP and pulse pressure indices.

## Introduction

Roughly one-seventh of the adult population worldwide exhibits elevated blood pressure (BP) levels, with more than half remaining oblivious to this condition^1^. Arterial hypertension (AHT) stands as the primary contributor to atherosclerotic cardiovascular disease (ASCVD) owing to the substantial number of patients who remain undiagnosed, undertreated, and inadequately monitored^2^.

It is well-established that an increase of 20 mmHg in systolic BP (SBP) and 10 mmHg in diastolic BP (DBP) is associated with a twofold increased risk of stroke and coronary artery disease (CAD)^3^. Moreover, a continuous and positive relationship was also observed between BP levels and a comprehensive spectrum of cardiovascular outcomes, encompassing hypertension-mediated organ damage (HMOD), subclinical ASCVD, coronary and stroke events, disability and mortality^4^. Recent research in ambulatory BP monitoring (ABPM) has established that this association may be stronger when considering nocturnal BP indices. Indeed, nocturnal SBP and DBP, day-night BP differences, and concerns surrounding the circadian BP profile have demonstrated an added prognostic value to 24-h, daytime, and office BP measurements for the risk of HMOD and cardiovascular events. Increased variability and BP load throughout the day, along with broader pulse pressure (PP) amplitudes, have also been linked to a compromised cardiovascular prognosis^5–8^.

Hypertensive patients often share several conditions that heighten the baseline risk of ASCVD, with lipid profile abnormalities being particularly noteworthy due to their prevalence and clinical significance^9^. Mendelian randomization studies and several clinical trials have consistently revealed a linear relationship between changes in plasma low-density lipoprotein cholesterol (LDL-c) levels and the risk of ASCVD. Additionally, an unfavorable triglyceride (TG)/high-density lipoprotein cholesterol (HDL-c) ratio, a prominent hallmark of the metabolic syndrome, has also been linked to high and very high ASCVD risk profiles^10,11^.

In contrast to conventional lipid markers, numerous investigations have identified lipoprotein (a) (Lp(a)) as an independent risk factor for ASCVD, including both CAD and stroke^12^. An estimated 5% of the population exhibits markedly elevated levels of Lp(a), which is in part a genetically determined risk factor for cardiovascular disease. Lipoprotein (a) is a low-density lipoprotein variant containing the specific apolipoprotein(a) (apo(a)) that infiltrates the subendothelium and binds atherogenic pro-inflammatory and oxidized phospholipids. This process triggers arterial inflammation and contributes to the development of arteriosclerosis. Additionally, due to its structural homology, apo(a) serves as a competitive inhibitor of plasminogen at its binding site, thereby facilitating thrombogenesis^13,14^.

The concurrent presence of elevated Lp(a) concentrations and increased BP levels has been documented in specific clinical studies and is substantiated by basic research^14–16^. Nevertheless, there is a lack of comprehensive understanding regarding the detailed profile of AHT based on Lp(a) levels, particularly concerning specific BP indices beyond office and diurnal BP measurements. Hence, we aimed to evaluate the relationship between Lp(a) concentrations and 24-h ABPM indices.

## Methods

### Study design, setting, and participants

This was an observational and cross-sectional study conducted in the Department of Internal Medicine at the University Hospital of Santiago de Compostela between June and December 2022. Patients aged 18 years and older, diagnosed with AHT, were enrolled. The initial screening for AHT was conducted using office BP measurements, with subsequent diagnostic confirmation obtained through the results of 24-h ABPM, in accordance with the European Society of Hypertension (ESH) guidelines^17^.

Clinical suspicion or confirmed diagnosis of secondary AHT, current smoking (within the 6 months prior to recruitment), and high-risk alcohol consumption (defined as more than 10 g and 20 g of alcohol per day for women and men, respectively) were exclusion criteria. Diabetes mellitus (DM) was defined in accordance with the American Diabetes Association (ADA) guidelines. Patients meeting the criteria for DM and those with clinical suspicion or confirmed diagnosis of primary hyperlipidemia were also excluded. All exclusion criteria are exposed in Supplementary Appendix 1^18,19^.

### Clinical baseline variables

Data collection encompassed demographic information including age and sex, along with lifestyle factors such as alcohol consumption (categorized as no versus low-risk drinking), former tobacco use (categorized as no/yes), and physical activity. Body mass index (BMI) was computed as the weight-to-height squared ratio, measured in Kg/m^2^. Waist circumference (WC) was measured above both iliac crests using a qualified tape measure and recorded in cm. The use of antihypertensive drugs was evaluated by therapeutic group, and adherence was assessed using the Morisky-Green questionnaire^17,20–22^.

### Parameters of 24-h ABPM collection

Patients underwent 24-h ABPM adhering to the STRIDE BP standards and using one of the following validated oscillometric devices: Space-Labs 90207 (Space-Labs Inc., Redmon, WA, USA), Microlife WatchBP O3 (Microlife Corporation, Widnau, Switzerland), and Cardioline Walk 200b (AB Medica Group S.A., Barcelona, Spain). Blood pressure levels were recorded every 20 min during the daytime and every 30 min during the night-time, and these periods of time were evaluated according to the patient's report. The test was considered reliable if more than 70% of the expected measurements were valid. On the day of testing, patients filled out a form regarding sleep times, medication usage, and any encountered concerns during recording^23,24^.

The following indices were obtained from the 24-h ABPM recordings: 24-h average SBP (24-hSBP), daytime SBP (dSBP), and night-time SBP (nSBP); 24-h average DBP (24-hDBP), daytime DBP (dDBP), and night-time DBP (nDBP). Night-time SBP decrease and nDBP dipping were computed as the percentage difference (ratio between (a) daytime index minus night-time index; and (b) daytime index) for each parameter. A non-dipper pattern was defined as a decrease in nSBP and / or nDBP of less than 10% compared with the average daytime values. A dipper pattern was defined as a decrease in nSBP and/or nDBP equal to or higher than 10% compared with the average daytime values. Pulse pressure was calculated as the systolic-diastolic BP gradient (SBP minus DBP) for each BP measurement. The values of 24-h PP (24-hPP), daytime PP (dPP), and night-time PP (nPP) were also collected and the unit of measurement was mmHg. Detailed data from BP variability and BP load are exposed in Supplementary Appendix 1^5,6,25^.

To assess the dynamic behavior of BP levels over time, we applied integral calculus using Simpson's method to compute the BP function. This approach allowed us to quantify the area under the function (AUF) for 24-h, daytime and night-time SBP (AUF 24-hSBP, AUF dSBP, AUF nSBP); DBP (AUF 24-hDBP, AUF dDBP, AUF nDBP), and PP (AUF 24-hPP, AUF dPP, AUF nPP). We also calculated the AUF for the nocturnal decrease in both SBP and DBP (AUF nSBP and AUF nDBP dipping) as the percentage difference (ratio between (a) daytime index minus night-time index; and (b) daytime index) for each parameter. The unit of measurement was cumulative mmHg within a given time period^26,27^.

### Laboratory variables

Blood samples were collected at 08:00 AM following a 12-h overnight fast and ensuring a minimum 12-h interval since the last use of antihypertensive medications and statins. After processing into serum, we conducted assays for fasting plasma glucose (FPG), uric acid, creatinine, total cholesterol (TC), TG, LDL-c, HDL-c and Lp(a) levels. Lipoprotein (a) levels were assessed through turbidimetry using a Binding Site Optilite system (The Binding Site Group Ltd., Birmingham, UK), with results expressed in nanomoles per liter (nmol/L). Detailed descriptions of the assay methodologies are available in Supplementary Appendix 1^28,29^.

### Ethical statement

This study was conducted in accordance with the ethical principles outlined in the Declaration of Helsinki and the standards of good practice (NBP) in research. Prior to participation, all patients were provided with comprehensive information about the study, ensuring their understanding and voluntary consent, and written informed consent was duly obtained. The study's protocol received formal approval from the Research Ethics Committee of Santiago-Lugo, underscoring our commitment to ethical standards in conducting biomedical research (code 2021/401, 19 October 2021).

### Statistical analysis

Statistical analysis was performed using SPSS 22.0 statistical software (SPSS Inc., Chicago, IL, USA). We conducted a descriptive analysis in which both qualitative and quantitative variables were expressed as number (percentage) and median (interquartile range), respectively. For univariate analysis, patients were categorized into two groups based on the Lp(a) level threshold of 125 nmol/L, as a cutoff derived from existing literature^16,23^. Qualitative and quantitative variables were compared using chi-squared and Mann–Whitney U tests, respectively. Upon identifying relevant associations between Lp(a) levels and BP indices, linear correlation analysis was undertaken, with results expressed as Spearman's correlation coefficients, to further explore these relationships. A p-value of less than 0.05 was set as the criterion for statistical significance.

Multivariate analysis was conducted using binary logistic regression, where the dependent variable was dichotomized Lp(a) levels (above or below 125 nmol/L). Predictor variables included BP indices that demonstrated statistical significance (p < 0.05) in the initial univariate analysis. Prior to constructing the maximum multivariate model for each BP index, a bivariate analysis was conducted using binary logistic regression to identify potential interactions with other covariates. Interaction terms were considered relevant if their coefficients reached statistical significance. Confounders were identified as variables altering the odds ratio by 10% or more. The selection of the optimal model for each BP index was guided by a comprehensive evaluation, including the omnibus test of model coefficients for initial validity, the Hosmer–Lemeshow test for goodness of fit, and adherence to binary logistic regression assumptions. Within the final models, only coefficients achieving statistical significance (p < 0.05) were considered relevant.

The sample size was calculated using Epidata^©^ software, available at http://www.epidata.dk/^30^. The calculations were based on the objective of detecting a standardized mean difference of 0.5 in the evaluated BP indices between the groups, as a common benchmark in many areas of biomedical research, often considered a "medium" effect size according to Cohen's conventions, with a 95% confidence level and a statistical power of at least 80%^31^.

## Results

### Descriptive and univariate analysis

#### General findings

We enrolled a cohort of 227 hypertensive patients, with a median age of 56 years, among whom 127 (56%) were women. Approximately one in five individuals reported low-risk alcohol consumption, while one in three were former smokers. Median values for BMI and WC fell within the overweight and abdominal obesity categories, respectively. In terms of hypertension management, all participants followed hygienic dietary advice, and nearly 80% were undergoing antihypertensive drug therapy, with a high level of adherence. In the context of therapeutic categories and ordered by frequency, the majority of patients received treatment with renin–angiotensin–aldosterone system (RAAS) blockers, followed by diuretics, calcium channel blockers (CCBs), and beta-blockers (BBs). Detailed results can be found in Table 1.

**Table 1:** Group comparisons of general findings based on the 75th percentile of lipoprotein(a) levels.

Variables	Total (n = 227)	Groups^a^		p-value
		Lp(a) ≤ 125 (nmol/L) n = 170	Lp(a) > 125 (nmol/L) n = 57	
Age (years)^e^	56 (17)	56 (17)	57 (17)	0.763
Sex (women)^f^	127 (56)	97 (57)	30 (53)	0.644
Alcohol intake^b,f^	42 (19)	31 (18)	11 (19)	0.846
Former smokers^c,f^	85 (37)	65 (38)	20 (35)	0.753
Physical activity^d,f^	95 (42)	76 (45)	19 (33)	0.162
Non-dipper (yes)^f^	97 (43)	68 (40)	29 (51)	0.165
BMI (Kg/m^2^)^e^	28 (7)	23 (7)	28 (7)	0.277
WC (cm)^e^	101 (19)	100 (20)	104 (13)	0.082
Adherence^f^	183 (81)	141 (83)	42 (74)	0.174
Antihypertensive drugs^f^	179 (79)	134 (79)	45 (79)	0.999
RAAS blockers^f^	128 (56)	93 (55)	35 (61)	0.441
Diuretics^f^	58 (26)	44 (26)	14 (25)	0.999
CCBs^f^	91 (42)	70 (43)	21 (37)	0.438
B-blockers^f^	35 (15)	26 (15)	9 (16)	0.999
Statins^f^	85 (37)	59 (35)	36 (46)	0.156
FPG (mg/dL)^e^	99 (17)	98 (16)	104 (18)	0.011
HbA1c (%)^e^	5.5 (0.4)	5.5 (0.5)	5.5 (0.4)	0.514
Creatinine (mg/dL)^e^	0.83 (0.3)	0.83 (0.3)	0.80 (0.3)	0.365
Uric acid (mg/dL)^e^	5.0 (2.4)	4.9 (2.4)	5.0 (2.3)	0.550
TG (mg/dL)^e^	92 (63)	91 (65)	94 (61)	0.367
TC (mg/dL)^e^	188 (47)	188 (47)	190 (49)	0.860
LDL-c (mg/dL)^e^	111 (39)	111 (38)	109 (43)	0.859
HDL-c (mg/dL)^e^	56 (22)	56 (23)	56 (18)	0.985
Lp(a) (nmol/L)^e^	46 (104)	35 (41)	191 (114)	< 0.001

*Lp(a)* lipoprotein(a), *BMI* body mass index, *WC* waist circumference, *RAAS* renin–angiotensin–aldosterone system, *CCBs* calcium channel blockers, *FPG* fasting plasma glucose, *HbA1c* glycosylated hemoglobin, *TG *triglycerides, *TC* total cholesterol, *LDL-c* low-density lipoprotein cholesterol, *HDL-c* high-density lipoprotein cholesterol, *kg* kilogram, *m* meter, *cm *centimeter, *mg* milligram, *dL* deciliter, *%* percentage, *nmol* nanomol, *L *liter.

^a^Patient groups according to the 75th percentile of Lp(a) levels.

^b^Low-risk alcohol consumption defined as less than 10 g and 20 g per day for women and men, respectively.

^c^Former smokers for more than 6 months at the time of recruitment.

^d^Moderate physical activity equivalent to 150 min of moderate-intensity walking per week.

^e^Results expressed in median and interquartile range.

^f^Results expressed in number and percentage.

After conducting a comparison between patients based on their Lp(a) levels, we observed no significant differences between the groups regarding demographic, epidemiological, clinical, and therapeutic variables. Both groups exhibited similar levels of adherence and frequency of antihypertensive drug use, as summarized in Table 1. The results of laboratory variables are extended in Supplementary Appendix 1.

#### Blood pressure indices

The median bedtime and wake-up time for both comparison groups were 11:00 PM and 08:00 AM hours, respectively. Overall, the median 24-h, daytime, and night-time SBP and DBP levels did not exceed the risk thresholds outlined in reference guidelines^17^. Notably, patients with Lp(a) concentrations above 125 nmol/L exhibited slightly elevated 24-h and night-time SBP levels, though these elevations did not reach statistical significance for DBP indices. The nocturnal decrease in SBP and DBP was at least 10% in all groups. Interestingly, patients with Lp(a) levels above 125 nmol/L exhibited a lower nDBP decrease, although this difference was not observed for nSBP dipping. Regarding PP, all indices, including nPP, were higher in patients with elevated Lp(a) concentrations. All results are detailed in Table 2.

**Table 2:** Comparison of blood pressure indices between groups based on the 75th percentile of lipoprotein(a) levels.

Variables	Total (n = 227)	Groups^a^		p-value
		Lp(a) ≤ 125 (nmol/L) (n = 170)	Lp(a) > 125 (nmol/L) (n = 57)	
24-hSBP (mmHg)^b^	125 (18)	123 (17)	130 (19)	0.025
dSBP (mmHg)^b^	129 (19)	127 (18)	132 (23)	0.053
nSBP (mmHg)^b^	114 (18)	111 (17)	120 (16)	0.004
nSBP dipping (%)^b^	11 (9)	12 (8)	10 (10)	0.112
24-hDBP (mmHg)^b^	77 (12)	77 (12)	76 (14)	0.943
dDBP (mmHg)^b^	80 (14)	80 (14)	78 (15)	0.729
nDBP (mmHg)^b^	67 (12)	67 (12)	70 (12)	0.126
nDBP dipping (%)^b^	15 (10)	16 (10)	13 (13)	0.011
24-hPP (mmHg)^b^	47 (12)	46 (11)	50 (11)	0.004
dPP (mmHg)^b^	47 (12)	47 (13)	51 (10)	0.005
nPP (mmHg)^b^	45 (11)	44 (11)	48 (11)	0.008

*Lp(a)* lipoprotein(a), *SBP* systolic blood pressure, *24-hSBP* average 24-h SBP, *dSBP* average daytime SBP, *nSBP* average night-time SBP, *DBP* diastolic blood pressure, *24-hDBP* average 24-h DBP, *dDBP* average daytime DBP, *nDBP* average night-time DBP, *PP* pulse pressure, *24-hPP* average 24-h PP, *dPP* average daytime PP, *nPP* average night-time PP, *mmHg* millimeter of mercury, *%* percentage.

^a^Groups attending to the 75th percentile of Lp(a) levels.

^b^Results expressed in median and interquartile range.

**Figure 1:**
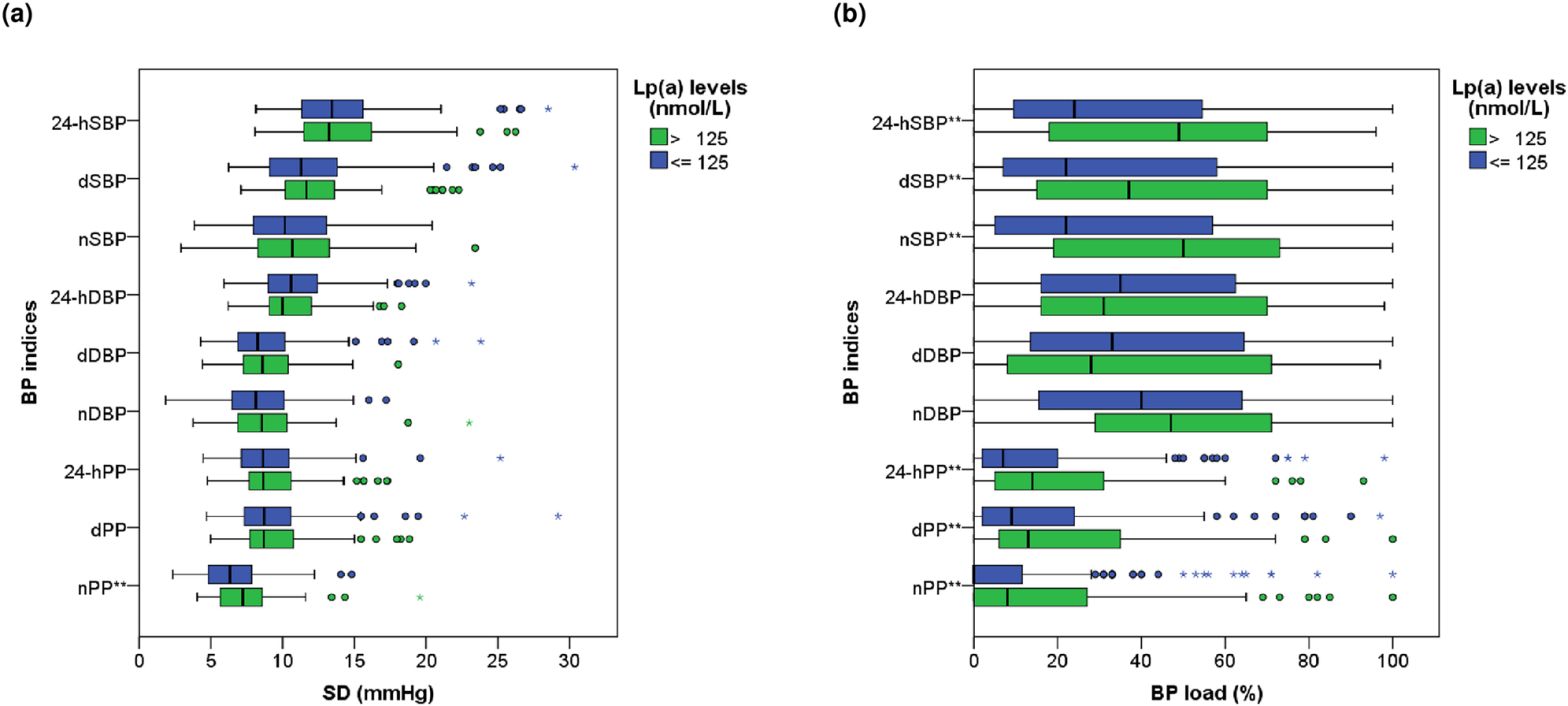
Comparison of (**a**) blood pressure variability and (**b**) blood pressure load between patients with Lp(a) levels below (blue) and above (green) 125 nmol/L. Results marked with double asterisk reached a p-value of less than 0.05. *Lp(a)* lipoprotein(a), *BP* blood pressure, *SD* standard deviation, *SBP* systolic blood pressure, *24-hSBP* average 24-h SBP, *dSBP* average daytime SBP, *nSBP* average night-time SBP, *DBP* diastolic blood pressure, *24-hDBP* average 24-h DBP, *dDBP* average daytime DBP, *nDBP* average night-time DBP, *PP* pulse pressure, *24-hPP* 24-h PP, *dPP* daytime PP, *nPP* night-time PP, *mmHg* millimeter of mercury, *nmol* nanomol, *L* liter, *%* percentage.

In terms of BP load, patients with elevated Lp(a) levels displayed significantly higher percentages of SBP and PP load throughout the 24-h period, daytime, and night-time. Nevertheless, no substantial disparities were observed concerning DBP indices. All findings are summarized in Supplementary Table 1(a). The results related to BP variability are commented in Supplementary Appendix 1 and detailed in Supplementary Table 1(b). Detailed graphical representations of BP load and BP variability can be found in Fig. 1.

**Figure 2:**
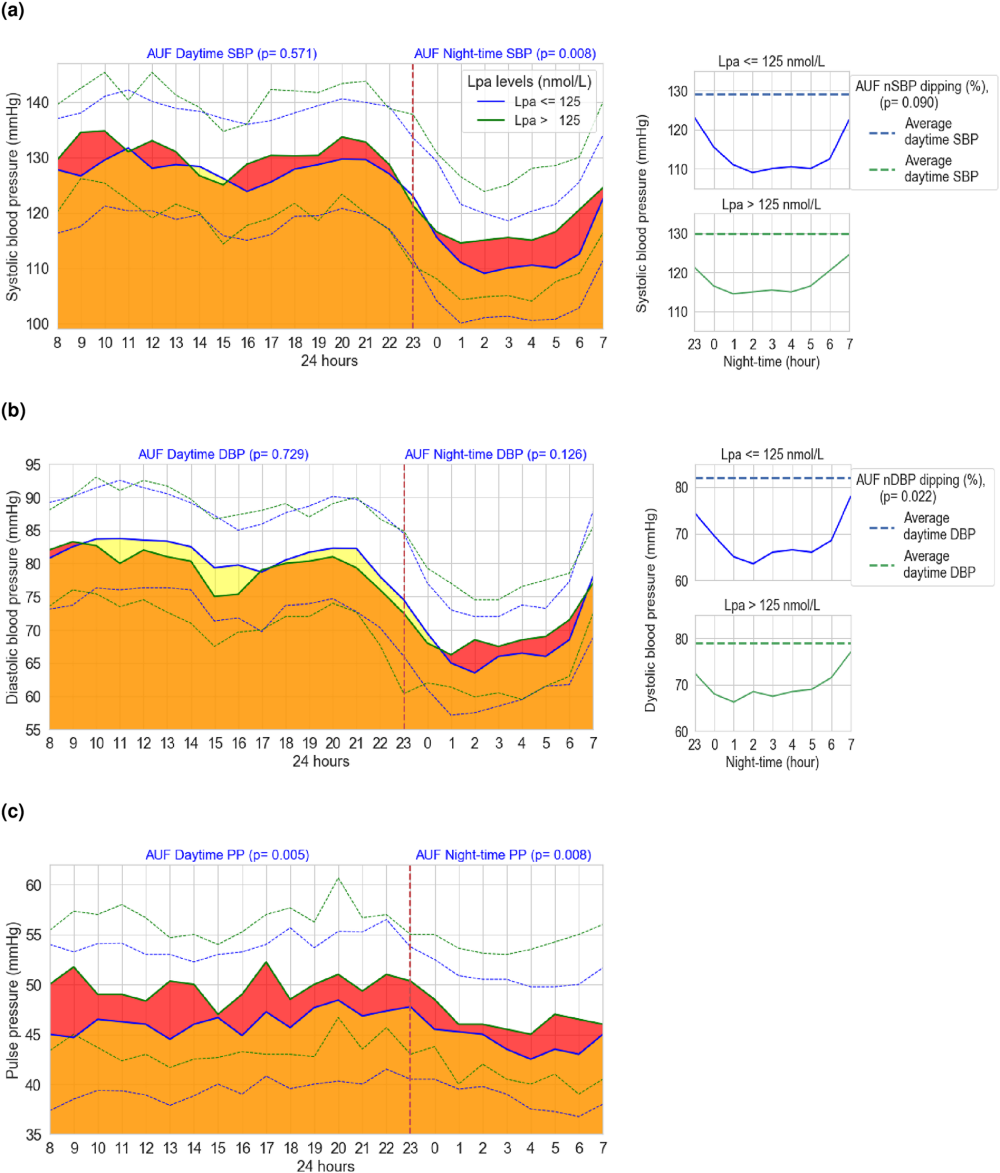
Dynamic behavioral analysis of blood pressure functions over time. Left side: this section depicts the BP functions over a 24-h period, including (**a**) systolic blood pressure, (**b**) diastolic blood pressure, and (**c**) pulse pressure. The vertical axis quantifies the evaluated BP indices, while the horizontal axis represents the time span. Solid lines illustrate the BP function over time in relation to Lp(a) concentrations (green for levels above and blue for levels at or below 125 nmol/L). Dotted lines indicate the interquartile ranges (IQR: Q3–Q1). The areas shaded down the solid lines represent the area under the function for BP levels. The red shaded region delineates periods where the value of the BP function in patients with elevated Lp(a) levels (> 125 nmol/L) surpassed that of patients with lower Lp(a) levels. Conversely, the pale yellow shaded area indicates intervals during which the value of the BP function of patients with lower Lp(a) levels surpassed that of patients with higher Lp(a) concentrations. Right side: this section illustrates the nocturnal BP features for (**a**) systolic blood pressure and (**b**) diastolic blood pressure, focusing on the nocturnal BP decline attending to Lp(a) levels (threshold of 125 nmol/L). The vertical axis shows the assessed BP index, while the horizontal axis denotes the nocturnal period. Dotted lines indicate the average daytime SBP and DBP (reference line), while solid lines illustrate the nocturnal BP function over time. Associated findings for Fig. 3 are detailed in Supplementary Tables 3. *BP* blood pressure, *Lp(a)* lipoprotein(a), *AUF* area under the function, *SBP* systolic blood pressure, *DBP* diastolic blood pressure, *nSBP* night-time SBP, *nDBP* night-time DBP, *mmHg* millimeter of mercury.

As depicted in Fig. 2 and Supplementary Table 1(c), the area under the BP function over time for various BP indices exhibited notable disparities among patients categorized by Lp(a) levels. Patients with elevated Lp(a) concentrations displayed higher AUF 24-hSBP and AUF nSBP values compared to their counterparts. In terms of nocturnal BP dipping, individuals with higher Lp(a) levels exhibited a larger AUF nDBP decrease, although this was not the case for AUF nSBP. As for PP, all indices (AUF 24-hPP, AUF dPP and AUF nPP) showed higher values in patients with Lp(a) levels above the cutoff point.

#### Linear correlation analysis

**Figure 3:**
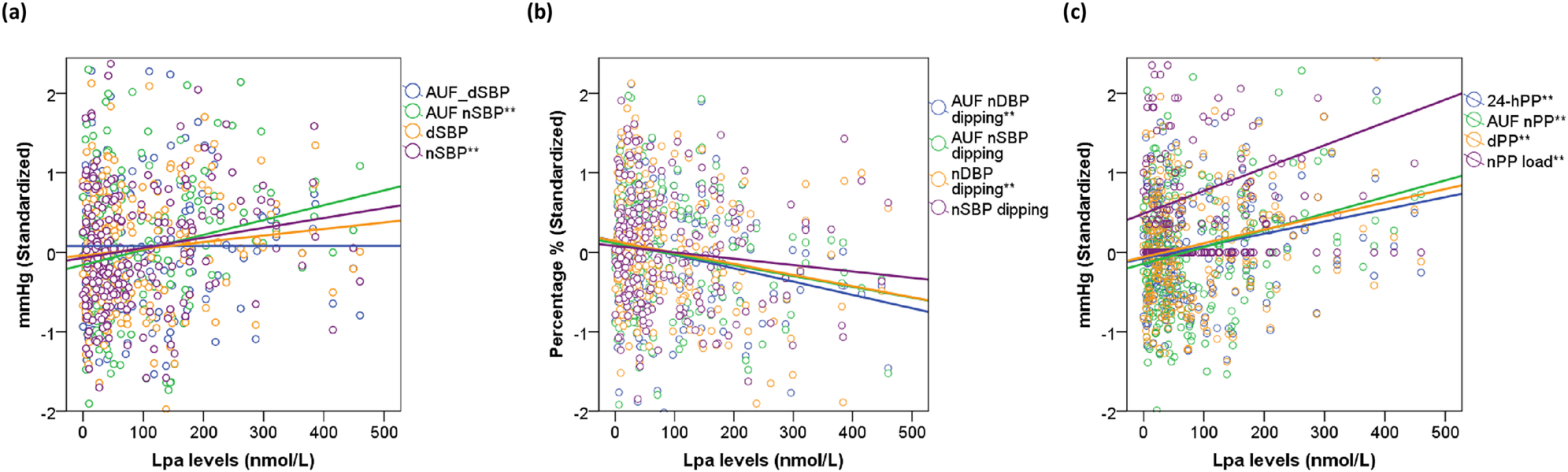
Results of linear correlation analysis for some indices of blood pressure. Correlation between Lp(a) levels and (**a**) systolic blood pressure indices, (**b**) systolic and diastolic nocturnal blood pressure dipping, (**c**) pulse pressure indices. Levels of BP indices were standardized by median and interquartile range. Results marked with asterisk reached a p-value less than 0.05. The complete results of the linear correlation analysis are presented in Supplementary Table 2a–d. *Lp(a)* lipoprotein (a), *BP* blood pressure, *AUF* area under the function, *SBP* systolic blood pressure, *dSBP* average daytime SBP, *nSBP* average night-time SBP, *AUF_dSBP* AUF of average daytime SBP, *AUF_nSBP* AUF of average night-time SBP, *DBP* diastolic blood pressure, *dDBP *average daytime DBP, *nDBP* average night-time DBP, *AUF_nDBP-AUF* of night-time DBP, *PP* pulse pressure, *24-hPP* 24-h PP, *AUF_24-hPP* AUF of 24-h pulse pressure, *dPP* daytime pulse pressure, *nPP* night-time pulse pressure, *mmHg* millimeter of mercury.

In addition to the disparities observed between the comparison groups, we identified weak positive linear correlations between Lp(a) concentrations and nSBP (Rho = 0.141, p = 0.034), 24 h-PP (Rho = 0.135, p = 0.043), dPP (Rho = 0.136, p = 0.041), along with a weak negative correlation with nDBP dipping (Rho = − 0.163, p = 0.014). Furthermore, a weak positive correlation was observed between Lp(a) levels and nPP load (Rho = 0.164, p = 0.013), while no significant findings were noted in relation to variability indices. Regarding the AUF of BP indices over time, weak positive correlations were found between Lp(a) levels and certain systolic and PP indices, with AUF nSBP (Rho = 0.140, p = 0.035) and AUF nPP (Rho = 0.151, p = 0.023) showing notable results, along with a weak negative correlation with AUF nDBP dipping (Rho = − 0.146, p = 0.028). The results can be found in Fig. 3 and are comprehensively outlined in Supplementary Tables 2(a–d).

### Multivariate analysis

The predictor variables that reached statistical significance (p < 0.05) were as follows: (1) for total BP indices, nSBP, nDBP dipping, and all PP indices (24-h PP, dPP, nPP); (2) for BP load, all systolic (24-h SBP, dSBP, nSBP) and PP (24-h PP, dPP, nPP) indices; and (3) for AUF of BP, AUF 24-hSBP, AUF nSBP, and all PP indices (AUF 24-hPP, AUF dPP, AUF nPP). The variables to be controlled during the analysis (p < 0.20) included adherence, statin use, physical activity, FPG, and WC. The final models for each predictor demonstrated both validity and goodness of fit. Due to pronounced collinearity among the BP indices, we eliminated additional BP indices in the model of each predictor.

Detailed descriptions of the final models for each predictor can be found in Table 3 and all data are provided in Supplementary Tables 3(a–o). The findings indicated that a 10 mmHg increase in nSBP was associated with more than 20% increased odds of belonging to the high Lp(a) level group. We also identified a protective interaction effect between adherence and nDBP dipping in the association between this predictor and Lp(a) levels. Subsequent stratified analysis revealed that each 1% increase in nDBP dipping was associated with a 12% decreased odds of high Lp(a) levels in patients with good adherence, while this effect was not observed in the other group (see Supplementary Fig. 1). Additionally, a 10 mmHg increase in all PP indices, including night-time PP, was also associated with more than a 40% heightened risk of high Lp(a) levels.

**Table 3:** Results of logistic regression models for the association between lipoprotein(a) levels and each blood pressure predictor.

Predictor	B	SE	p-value	Exp(B)	CI95% (lower–upper)
Average BP					
nSBP (mmHg)	0.023	0.011	0.044	1.0230	1.0010–1.0450
nDBP dipping (by) adherence (%)	–0.118	0.049	0.017	0.8890	0.8070–0.9790
24-hPP (mmHg)	0.047	0.018	0.011	1.0480	1.0110–1.0860
dPP (mmHg)	0.046	0.017	0.008	1.0470	1.0120–1.0830
nPP (mmHg)	0.044	0.018	0.016	1.0440	1.0080–1.0820
BP load					
24-hSBP load (%)	0.012	0.005	0.025	1.0120	1.0020–1.0230
dSBP load (%)	0.010	0.005	0.046	1.0100	1.0002–1.0203
nSBP load (%)	0.011	0.005	0.019	1.0110	1.0020–1.0210
24-hPP load (%)	0.015	0.007	0.048	1.0150	1.0001–1.0295
nPP load (%)	0.017	0.007	0.013	1.0170	1.0040–1.0310
AUF of BP					
AUF nSBP (mmHg)	0.002	0.001	0.047	1.0021	1.0001–1.0042
AUF nDBP dipping (%)	–0.034	0.017	0.045	0.9660	0.9350–0.9990
AUF 24–hPP (mmHg)	0.002	0.001	0.015	1.0020	1.0001–1.0039
AUF dPP (mmHg)	0.002	0.001	0.045	1.0020	1.0001–1.0042
AUF nPP (mmHg)	0.005	0.002	0.019	1.0046	1.0001–1.0085

Logistic regression models elucidating the relationship between blood pressure indices (predictor variables) and Lp(a) levels are presented. All models incorporated data from 227 patients, ensuring no missing data. The variables to be controlled were adherence, statin use, physical activity, FPG, and WC. Only blood pressure indices demonstrating statistical significance (p < 0.05) are showed. All comprehensive models including those variables within the final model are detailed in Supplementary Tables 3a–o.

*SBP* systolic blood pressure, *nSBP* average night-time SBP, *DBP* diastolic blood pressure, *nDBP* average night-time DBP, *PP* pulse pressure, *24-hPP* average 24-hour PP, *dPP* average daytime PP, *nPP* average night-time PP, *24-hSBP* average 24-hour SBP, *dSBP* average daytime SBP, *AUF* area under the function, *mmHg* millimeter of mercury, *%* percentage.

In terms of BP load, the results revealed that a 10-point percentage rise in 24-h nSBP, dSBP, and nSBP load was associated with at least a 10% higher likelihood of being in the high Lp(a) group. Similarly, a 10-point percentage increase in 24-h PP and nPP load resulted in more than a 15% higher odds ratio of being in the high Lp(a) group (as presented in Table 3). The results related to BP variability did not reach statistical significance.

As for the area under the function of BP levels over time, we found that a 100 cumulative mmHg rise in AUF nSBP was associated with more than a 20% increase in the odds of presenting elevated Lp(a) levels, whereas each 10% increase in AUF nDBP dipping was associated with more than a 30% decrease in the odds of belonging to the elevated Lp(a) levels category. We also found that a 100 mmHg increase in AUF 24-hPP and AUF dPP was associated with a 20% greater risk of high Lp(a) levels. However, when focusing on nPP, we observed that a 100 mmHg increase was associated with an almost 50% greater risk of elevated Lp(a) levels.

## Discussion

The current study reveals an association between Lp(a) levels and several night-time BP indices, among others. The key findings can be summarized as follows: (1) Individuals with high Lp(a) levels exhibited increased nocturnal systolic BP levels and reduced night-time diastolic BP dipping; (2) There was a positive association between night-time pulse pressure and Lp(a) concentrations; (3) Elevated Lp(a) levels were also associated with higher night-time systolic BP and pulse pressure load; (4) patients with elevated Lp(a) levels displayed a greater area under the function over time for nocturnal systolic BP and pulse pressure indices, with a smaller area under the function over time for diastolic BP dipping, compared to those with lower Lp(a) levels.

These findings align with the study's objectives, supporting that Lp(a) levels are associated not only with higher overall BP levels, as shown in previous research^32–34^. From a pathogenic perspective, the results are also consistent with those from several Mendelian randomization studies which have unveiled a polygenic relationship between Lp(a) levels and BP. Furthermore, these findings harmonize with data from other basic research that associate Lp(a) levels with oxidative stress, inflammation and atherosclerotic vascular damage^35,36^. Despite this, clinical understanding of the Lp(a)-BP relationship, especially during night-time, remains somewhat underexplored. Real-world clinical studies examining the Lp(a)-BP nexus are scarce and often plagued by small cohorts, absence of control groups, and a focus primarily on conventional BP measurements^37–39^.

As known from other BP indices, increased night-time BP levels along with a blunted nocturnal BP decline have been associated with vasomotor regulation abnormalities. A main feature in vasomotor tone dysregulation is endothelial dysfunction, frequently related to a detrimental lipid profile. In this sense, elevated levels of Lp(a) have been established as a critical pro-oxidant and pro-inflammatory insult within the vascular wall, promoting endothelial dysfunction. Consequently, these heightened Lp(a) levels may be also linked to specific nocturnal BP abnormalities^40–42^.

The potential protective influence of adherence on the association between nDBP decrease and Lp(a) levels supports that some antihypertensive and lipid-lowering drugs may exert pleiotropic effects, influencing hemodynamics, as has been suggested by previous studies. However, These findings should be interpreted with caution, as the study's primary objective did not encompass exploring this relationship, and the methodology was not specifically designed for this purpose^43,44^.

Aging is linked to a progressive rise in SBP along and a decline in DBP, leading to increased PP amplitude. Given these findings and no age differences between groups, elevated nSBP levels along with greater PP amplitudes in individuals with high Lp(a), may indicate premature vascular aging, as suggested by prior research. Pulse pressure serves as an indicator of indirect vascular HMOD, providing insights into the stiffness of central arteries. Elevated PP levels are associated with poorer cardiovascular outcomes, stemming from elevated peak systolic BP and critical diastolic hypoperfusion of vital organs, particularly the heart and the central nervous system^34,45,46^.

The prognostic relevance of nPP levels is becoming increasingly well known. Nevertheless, the connection between Lp(a) levels and nPP remains an underexplored field. This situation could have several readings, as elevated Lp(a) levels and wide PP are concurrent phenomena in vascular damage, but may also exhibit a dual relationship according to prior research. Beyond its independent association with arterial stiffness and critical hypoperfusion of diastolic flow-dependent organs, recent studies have linked nPP to a pro-inflammatory internal milieu and an unfavorable redox status. These abnormalities promote a sequence of deleterious metabolic effects on the cardiovascular system, potentially including concurrent lipid profile abnormalities^47–49^.

Blood pressure load has also been associated with arterial stiffness and cumulative burden of vascular damage, beyond the average values of conventional BP indices. This index provides us with insights into the time under high BP levels and wide PP amplitudes, which carries prognostic significance as established in previous studies. Besides shedding light on daytime BP load, this study underscores a stronger association between nocturnal BP load and Lp(a) levels^50,51^.

While average BP readings offer a general snapshot of the Lp(a)-BP relationship, analyzing the dynamic fluctuations of BP over time provides a more detailed understanding. This method yielded both visual and analytical data backing the primary hypothesis and aligning with previous research. This approach support that Lp(a) levels are linked not only to average BP levels but also with the cumulative BP over time, hinting that the BP "tsunami" may be potentially as relevant as the "wave" that precedes it.

Previous research has established a connection between Lp(a) concentrations, BP levels, and atherosclerotic cardiovascular disease. Furthermore, nocturnal BP indices, pulse pressure and BP load, among others, have demonstrated an important prognostic value beyond traditional BP measurements. This study has shown that the association between Lp(a) and BP levels may extend beyond conventional office and diurnal BP measurements, encompassing additional BP indices critical for understanding the magnitude of cardiovascular risk due to elevated BP. Routine evaluation of lipoprotein(a) in hypertensive patients, especially those displaying abnormalities in the aforementioned indices, may contribute to improved stratification of cardiovascular risk, leading to more effective decision-making.

### Strengths and limitations

The cross-sectional design of the study inherently limits its scope, while the specific inclusion and exclusion criteria, along with the small sample size achieved, may constrain its external validity. While a wide range of variables were examined, there might be unconsidered factors that could influence Lp(a) levels and/or BP measurements. Despite the daily variability of some laboratory values, including lipid profiles, Lp(a) levels tend to remain stable. The 24-h ABPM indices can fluctuate daily under different conditions, so standardizing ABPM according to major protocols should help mitigate this possible bias. Analyzing the complex interrelations among BP indices is challenging, as they are often interconnected and functionally related. To reduce inaccuracies from multicollinearity, we analyzed BP indices separately and in relation to other confounding and/or interacting variables. Despite these limitations, the study was developed in a real clinical setting employing robust methodological approaches, and included a comprehensive assessment of lipid profile and 24-h ABPM data from a specific and well-featured patient cohort.

### Supplementary Information

Supplementary Information.

## Data Availability

Data presented in this study are available on request from the corresponding author. In accordance with Article 18.4 of the Spanish Constitution and the Organic Law on Data Protection and Guarantee of Digital Rights (LOPDGDD) of 6 December 2018, the privacy and integrity of the individual will be protected at all times, so anonymous data are available upon reasonable request.
