# Supplementary material for: Assessing the relationship between lipoprotein(a) levels and blood pressure among hypertensive patients beyond conventional measures. An observational study
Source: Sci Rep. 2024 Jun 23;14:14433. doi: 10.1038/s41598-024-65231-w (PMC11194270; doi:10.1038/s41598-024-65231-w)
Supplement: Supplementary file 1 — Supplementary Information. [file 41598_2024_65231_MOESM1_ESM.zip › Supplementary Tables 1.docx]

***Supplementary Tables 1. Univariate comparisons***

**(a) Comparison of blood pressure load between groups based on the 75th percentile of lipoprotein (a) levels.**

| **Variables** | **Total** | **Groups^a^** | | **p-value** |
| --- | --- | --- | --- | --- |
|  | **n= 227** | **Lp(a) ≤ 125 (nmol/L)**  **n= 170** | **Lp(a) > 125 (nmol/L)**  **n= 57** |  |
| 24-hSBP (%) † | 32 (51) | 24 (46) | 49 (53) | 0.014 |
| dSBP (%) † | 29 56) | 23 (52) | 37 (56) | 0.026 |
| nSBP (%) † | 25 (58) | 22 (52) | 50 (56) | 0.010 |
| 24-hDBP (%) † | 34 (51) | 35 (48) | 31 (55) | 0.907 |
| dDBP (%) † | 32 (55) | 33 (52) | 28 (64) | 0.669 |
| nDBP (%) † | 41 (49) | 40 (49) | 47 (45) | 0.118 |
| 24-hPP (%) † | 9 (22) | 7 (18) | 14 (28) | 0.019 |
| dPP (%) † | 10 (26) | 9 (22) | 13 (30) | 0.037 |
| nPP (%) † | 0 (17) | 0 (12) | 8 (28) | 0.001 |

^a^ Groups attending to the 75th percentile of Lp(a) levels. Results expressed as † refer to the median and interquartile range. Lp(a)—Lipoprotein (a); SBP—Systolic blood pressure; 24-hSBP—Average 24-hour SBP; dSBP—Average daytime SBP; nSBP—Average night-time SBP; DBP—Diastolic blood pressure; 24-hDBP—Average 24-hour DBP; dDBP—Average daytime DBP; nDBP—Average night-time DBP; PP—Pulse pressure; 24-hPP—Average 24-hour PP; dPP—Average daytime PP; nPP—Average night-time PP; %—Percentage.

**(b) Comparison of blood pressure variability between groups based on the 75th percentile of lipoprotein (a) levels.**

| **Variables** | **Total** | **Groups^a^** | | **p-value** |
| --- | --- | --- | --- | --- |
|  | **n= 227** | **Lp(a) ≤ 125 (nmol/L)**  **n= 170** | **Lp(a) > 125 (nmol/L)**  **n= 57** |  |
| 24-hSBP (mmHg) † | 13 (5) | 13.5 (4) | 13.2 (5) | 0.939 |
| dSBP (mmHg) † | 11 (5) | 11.3 (5) | 11.7 (4) | 0.255 |
| nSBP (mmHg) † | 10 (5) | 10.1 (5) | 10.7 (5) | 0.353 |
| 24-hDBP (mmHg) † | 10 (3) | 10.8(3) | 10.0 (3) | 0.514 |
| dDBP (mmHg) † | 8 (3) | 8.3 (3) | 8.6 (3) | 0.362 |
| nDBP (mmHg) † | 8 (4) | 8.1 (4) | 8.6 (4) | 0.311 |
| 24-hPP (mmHg) † | 9 (3) | 8.6 (3) | 8.6 (3) | 0.352 |
| dPP (mmHg) † | 9 (3) | 8.7 (3) | 8.7 (3) | 0.689 |
| nPP (mmHg) † | 6 (3) | 6.3 (3) | 7.2 (3) | 0.016 |

^a^ Groups attending to the 75th percentile of Lp(a) levels. Results expressed as † refer to the median and interquartile range. Lp(a)—Lipoprotein (a); SBP—Systolic blood pressure; 24-hSBP—Average 24-hour SBP; dSBP—Average daytime SBP; nSBP—Average night-time SBP; DBP—Diastolic blood pressure; 24-hDBP—Average 24-hour DBP; dDBP—Average daytime DBP; nDBP—Average night-time DBP; PP—Pulse pressure; 24-hPP—Average 24-hour PP; dPP—Average daytime PP; nPP—Average night-time PP; mmHg—Millimeter of mercury.

**(c) Comparison of the area under the function of BP indices over time according to Lp(a) levels.**

| **Variables^b^** | **Total** | **Groups^a^** | | **p-value** |
| --- | --- | --- | --- | --- |
|  | **n= 227** | **Lp(a) ≤ 125 (nmol/L)**  **n= 170** | **Lp(a) > 125 (nmol/L)**  **n= 57** |  |
| AUF_24-hSBP (mmHg) † | 2849 (429) | 2821 (405) | 2955 (449) | 0.023 |
| AUF_dSBP (mmHg) † | 1945 (337) | 1935 (337) | 1960 (350) | 0.571 |
| AUF_nSBP (mmHg) † | 853 (177) | 840 (171) | 916 (184) | 0.008 |
| AUF_nSBP dipping (%) † | 56 (12) | 56 (11) | 54 (11) | 0.090 |
| AUF_24-hDBP (mmHg) † | 1764 (295) | 1765 (283) | 1734 (330) | 0.957 |
| AUF_dDBP (mmHg) † | 1224 (222) | 1230 (2188) | 1195 (252) | 0.290 |
| AUF_nDBP (mmHg) † | 529 (112) | 512 (114) | 551 (100) | 0.060 |
| AUF_nDBP dipping (%) † | 57 (12) | 58 (12) | 55 (11) | 0.022 |
| AUF_24-hPP (mmHg) † | 1079 (273) | 1063 (259) | 1153 (242) | 0.005 |
| AUF_dPP (mmHg) † | 725 (191) | 718 (201) | 770 (161) | 0.028 |
| AUF_nPP (mmHg) † | 355 (88) | 346 (87) | 379 (87) | 0.007 |

^a^ Groups attending to the 75th percentile of Lp(a) levels. Results expressed as † refer to the median and interquartile range. ^b^ AUF—Area under the function (calculated as the integral of the function by Simpson's method). Lp(a)—Lipoprotein (a); SBP—Systolic blood pressure; AUF_24-hSBP—AUF of 24-hour SBP; AUF_dSBP—AUF of daytime SBP; AUF_nSBP—AUF of night-time SBP; DBP—Diastolic blood pressure; AUF_24-hDBP—AUF of 24-hour DBP; AUF_dDBP—AUF of daytime DBP; AUF_nDBP—AUF of night-time DBP; PP—Pulse pressure; AUF_24-hPP—AUF of 24-hour PP; AUF_dPP—AUF of daytime PP; AUF_nPP—AUF of night-time PP; mmHg—Millimeter of mercury; %—Percentage.
