# Supplementary material for: Assessing the relationship between lipoprotein(a) levels and blood pressure among hypertensive patients beyond conventional measures. An observational study
Source: Sci Rep. 2024 Jun 23;14:14433. doi: 10.1038/s41598-024-65231-w (PMC11194270; doi:10.1038/s41598-024-65231-w)
Supplement: Supplementary file 1 — Supplementary Information. [file 41598_2024_65231_MOESM1_ESM.zip › Supplementary Tables 3.docx]

***Supplementary Tables 3. Binary logistic regression models for Lp(a) levels on the basis of BP indices.***

1. **Night-time systolic blood pressure**

| **Variables** | **B** | **SE** | **p-value** | **Exp(B)** | **CI 95% (lower–upper)** | |
| --- | --- | --- | --- | --- | --- | --- |
| Physical activity | -0.350 | 0.332 | 0.293 | 0.705 | 0.368 | 1.352 |
| Adherence (yes) | -0.668 | 0.385 | 0.083 | 0.513 | 0.241 | 1.091 |
| Statin use (yes) | 0.492 | 0.330 | 0.137 | 1.635 | 0.855 | 3.124 |
| FPG (mg/dL) | 0.016 | 0.010 | 0.107 | 1.016 | 0.997 | 1.035 |
| nSBP (mmHg) | 0.023 | 0.011 | 0.044 | 1.0230 | 1.0010 | 1.0450 |
| B_0_ | -4.845 | 1.647 | 0.003 | 0.008 |  | |

Logistic regression model elucidating the relationship between nSBP and Lp(a) levels are presented. The model incorporated data from 227 patients. ensuring no missing data. The variables to be controlled were adherence. statin use. physical activity. FPG. and WC. Omnibus test of model coefficients (p-value) = 0.012. Hosmer-Lemeshow test (p-value) = 0.547.

**b) Night-time diastolic blood pressure dipping**

| **Variables** | **B** | **SE** | **p-value** | **Exp(B)** | **CI 95% (lower–upper)** | |
| --- | --- | --- | --- | --- | --- | --- |
| nDBP dipping (by) adherence (%) | -0.118 | 0.049 | 0.017 | 0.8890 | 0.8070 | 0.9790 |
| nDBP dipping (%) | 0.031 | 0.042 | 0.464 | 1.031 | 0.950 | 1.119 |
| Adherence (yes) | 1.017 | 0.858 | 0.236 | 2.764 | 0.514 | 14.864 |
| Statin use (yes) | 0.514 | 0.336 | 0.126 | 1.672 | 0.866 | 3.230 |
| FPG (mg/dL) | 0.018 | 0.010 | 0.062 | 1.018 | 0.999 | 1.038 |
| B_0_ | -3.131 | 1.333 | 0.019 | 0.044 |  |  |

Logistic regression model elucidating the relationship between nDBP dipping and Lp(a) levels are presented. The model incorporated data from 227 patients. ensuring no missing data. The variables to be controlled were adherence. statin use. physical activity. FPG. and WC. Omnibus test of model coefficients (p-value) = 0.001. Hosmer-Lemeshow test (p-value) = 0.109.

1. **24-hour pulse pressure**

| **Variables** | **B** | **SE** | **p-value** | **Exp(B)** | **CI 95% (lower–upper)** | |
| --- | --- | --- | --- | --- | --- | --- |
| 24-hPP (mmHg) | 0.047 | 0.018 | 0.010 | 1.0480 | 1.0110 | 1.0860 |
| Adherence (yes) | -0.807 | 0.387 | 0.037 | 0.446 | 0.209 | 0.952 |
| Statin use (yes) | 0.451 | 0.332 | 0.174 | 1.570 | 0.820 | 3.006 |
| FPG (mg/dL) | 0.016 | 0.010 | 0.087 | 1.017 | 0.998 | 1.036 |
| B_0_ | -4.615 | 1.296 | < 0.001 | 0.010 |  | |

Logistic regression model elucidating the relationship between 24-hPP and Lp(a) levels are presented. The model incorporated data from 227 patients. ensuring no missing data. The variables to be controlled were adherence. statin use. physical activity. FPG. and WC. Omnibus test of model coefficients (p-value) = 0.003. Hosmer-Lemeshow test (p-value) = 0.224.

1. **Daytime pulse pressure**

| **Variables** | **B** | **SE** | **p-value** | **Exp(B)** | **CI 95% (lower–upper)** | |
| --- | --- | --- | --- | --- | --- | --- |
| dPP (mmHg) | 0.046 | 0.017 | 0.008 | 1.0470 | 1.0120 | 1.0830 |
| Adherence (yes) | -0.827 | 0.388 | 0.033 | 0.437 | 0.204 | 0.937 |
| Statin use (yes) | 0.469 | 0.332 | 0.157 | 1.599 | 0.835 | 3.062 |
| FPG (mg/dL) | 0.016 | 0.010 | 0.095 | 1.016 | 0.997 | 1.035 |
| B_0_ | -4.531 | 1.265 | < 0.001 | 0.011 |  | |

Logistic regression model elucidating the relationship between dPP and Lp(a) levels are presented. The model incorporated data from 227 patients. ensuring no missing data. The variables to be controlled were adherence. statin use. physical activity. FPG. and WC. Omnibus test of model coefficients (p-value) = 0.003. Hosmer-Lemeshow test (p-value) = 0.178.

1. **Night-time Pulse pressure**

| **Variables** | **B** | **SE** | **p-value** | **Exp(B)** | **CI 95% (lower–upper)** | |
| --- | --- | --- | --- | --- | --- | --- |
| nPP (mmHg) | 0.044 | 0.018 | 0.016 | 1.0440 | 1.0080 | 1.0820 |
| Adherence (yes) | -0.615 | 0.373 | 0.099 | 0.541 | 0.260 | 1.123 |
| FPG (mg/dL) | 0.018 | 0.009 | 0.052 | 1.019 | 1.000 | 1.038 |
| B_0_ | -4.561 | 1.301 | < 0.001 | 0.010 |  |  |

Logistic regression model elucidating the relationship between nSBP and Lp(a) levels are presented. The model incorporated data from 227 patients. ensuring no missing data. The variables to be controlled were adherence. statin use. physical activity. FPG. and WC. Omnibus test of model coefficients (p-value) = 0.006. Hosmer-Lemeshow test (p-value) = 0.111.

1. **24-hour systolic blood pressure load**

| **Variables** | **B** | **SE** | **p-value** | **Exp(B)** | **CI 95% (lower–upper)** | |
| --- | --- | --- | --- | --- | --- | --- |
| 24-hSBP load (%) | 0.012 | 0.005 | 0.025 | 1.0120 | 1.0020 | 1.0230 |
| Adherence (yes) | -0.694 | 0.384 | 0.071 | 0.499 | 0.235 | 1.060 |
| Statin use (yes) | 0.519 | 0.330 | 0.116 | 1.680 | 0.880 | 3.207 |
| FPG (mg/dL) | 0.017 | 0.010 | 0.083 | 1.017 | 0.998 | 1.036 |
| B_0_ | -2.934 | 1.038 | 0.005 | 0.053 |  | |

Logistic regression model elucidating the relationship between nSBP and Lp(a) levels are presented. The model incorporated data from 227 patients. ensuring no missing data. The variables to be controlled were adherence. statin use. physical activity. FPG. and WC. Omnibus test of model coefficients (p-value) = 0.007. Hosmer-Lemeshow test (p-value) = 0.584.

1. **Daytime systolic blood pressure load**

| **Variables** | **B** | **SE** | **p-value** | **Exp(B)** | **CI 95% (lower–upper)** | |
| --- | --- | --- | --- | --- | --- | --- |
| dSBP load (%) | 0.010 | 0.005 | 0.046 | 1.0100 | 1.0002 | 1.0200 |
| Adherence (yes) | -0.693 | 0.383 | 0.070 | 0.500 | 0.236 | 1.059 |
| Statin use (yes) | 0.527 | 0.329 | 0.109 | 1.693 | 0.889 | 3.226 |
| FPG (mg/dL) | 0.017 | 0.010 | 0.076 | 1.017 | 0.998 | 1.036 |
| B_0_ | -2.901 | 1.036 | 0.005 | 0.055 |  |  |

Logistic regression model elucidating the relationship between nSBP and Lp(a) levels are presented. The model incorporated data from 227 patients. ensuring no missing data. The variables to be controlled were adherence. statin use. physical activity. FPG. and WC. Omnibus test of model coefficients (p-value) = 0.011. Hosmer-Lemeshow test (p-value) = 0.743.

1. **Night-time systolic blood pressure load**

| **Variables** | **B** | **SE** | **p-value** | **Exp(B)** | **CI 95% (lower–upper)** | |
| --- | --- | --- | --- | --- | --- | --- |
| nSBP load (%) | 0.011 | .005 | 0.019 | 1.0110 | 1.0020 | 1.0210 |
| Adherence (yes) | -0.656 | 0.386 | 0.089 | 0.519 | 0.244 | 1.105 |
| Statin use (yes) | 0.485 | 0.331 | 0.143 | 1.624 | 0.849 | 3.104 |
| FPG (mg/dL) | 0.016 | 0.010 | 0.096 | 1.016 | 0.997 | 1.036 |
| B_0_ | -2.855 | 1.033 | 0.006 | 0.058 |  | |

Logistic regression model elucidating the relationship between nSBP and Lp(a) levels are presented. The model incorporated data from 227 patients. ensuring no missing data. The variables to be controlled were adherence. statin use. physical activity. FPG. and WC. Omnibus test of model coefficients (p-value): 0.006. Hosmer-Lemeshow test (p-value)= 0.121.

1. **24-hour pulse pressure load**

| **Variables** | **B** | **SE** | **p-value** | **Exp(B)** | **CI 95% (lower-upper)** | |
| --- | --- | --- | --- | --- | --- | --- |
| 24-hPP load (%) | 0.015 | 0.007 | 0.048 | 1.0150 | 1.0001 | 1.0295 |
| Adherence (yes) | -0.767 | .383 | 0.045 | .464 | 0.219 | .984 |
| Statin use (yes) | 0.466 | 0.330 | 0.157 | 1.594 | 0.835 | 3.041 |
| FPG (mg/dL) | 0.017 | 0.010 | 0.079 | 1.017 | 0.998 | 1.036 |
| B_0_ | -2.666 | 1.020 | .009 | 0.070 |  |  |

Logistic regression model elucidating the relationship between nSBP and Lp(a) levels are presented. The model incorporated data from 227 patients. ensuring no missing data. The variables to be controlled were adherence. statin use. physical activity. FPG. and WC. Omnibus test of model coefficients (p-value) = 0.011. Hosmer-Lemeshow test (p-value) = 0.968.

1. **Night pulse pressure load**

| **Variables** | **B** | **SE** | **p-value** | **Exp(B)** | **CI 95% (lower-upper)** | |
| --- | --- | --- | --- | --- | --- | --- |
| nPP load (%) | 0.017 | 0.007 | 0.013 | 1.0170 | 1.0040 | 1.0310 |
| Adherence (yes) | -0.693 | 0.384 | 0.071 | 0.500 | 0.236 | 1.061 |
| Statin use (yes) | 0.426 | 0.333 | 0.201 | 1.531 | 0.797 | 2.939 |
| FPG (mg/dL) | 0.017 | 0.010 | 0.073 | 1.017 | 0.998 | 1.037 |
| B_0_ | -2.734 | 1.030 | 0.008 | 0.065 |  | |

Logistic regression model elucidating the relationship between nSBP and Lp(a) levels are presented. The model incorporated data from 227 patients. ensuring no missing data. The variables to be controlled were adherence. statin use. physical activity. FPG. and WC. Omnibus test of model coefficients (p-value) = 0.004. Hosmer-Lemeshow test (p-value) = 0.570.

1. **Area under the function of night-time systolic blood pressure**

| **Variables** | **B** | **SE** | **p-value** | **Exp(B)** | **CI 95% (lower-upper)** | |
| --- | --- | --- | --- | --- | --- | --- |
| AUF nSBP (mmHg) | 0.002 | 0.001 | 0.047 | 1.0021 | 1.0001 | 1.0042 |
| FPG (mg/dL) | 0.016 | 0.010 | 0.088 | 1.016 | 0.998 | 1.036 |
| Statin use (yes) | 0.349 | 0.320 | 0.275 | 1.418 | 0.758 | 2.652 |
| B_0_ | -4.764 | 1.318 | <0.001 | 0.009 |  | |

Logistic regression model elucidating the relationship between nSBP and Lp(a) levels are presented. The model incorporated data from 227 patients. ensuring no missing data. The variables to be controlled were adherence. statin use. physical activity. FPG. and WC. Omnibus tests of model coefficients (p-value): 0.019. Hosmer-Lemeshow test (p-value)= 0.400.

1. **Area under the function for night-time diastolic blood pressure dipping**

| **Variables** | **B** | **SE** | **p-value** | **Exp(B)** | **CI 95% (lower-upper)** | |
| --- | --- | --- | --- | --- | --- | --- |
| Physical activity | -0.447 | 0.333 | 0.179 | 0.639 | 0.333 | 1.228 |
| Adherence (yes) | -0.709 | 0.385 | 0.065 | 0.492 | 0.232 | 1.046 |
| Statin use (yes) | 0.475 | 0.331 | 0.151 | 1.607 | 0.840 | 3.074 |
| FPG (mg/dL) | 0.015 | 0.010 | 0.119 | 1.015 | 0.996 | 1.035 |
| AUF nDBP dipping (%) | -0.034 | 0.017 | 0.045 | 0.9660 | 0.9350 | 0.9990 |
| B_0_ | -0.205 | 1.488 | 0.890 | 0.815 |  |  |

Logistic regression model elucidating the relationship between nSBP and Lp(a) levels are presented. The model incorporated data from 227 patients. ensuring no missing data. The variables to be controlled were adherence. statin use. physical activity. FPG. and WC. Omnibus test of model coefficients (p-value) = 0.012. Hosmer-Lemeshow test (p-value) = 0.152.

1. **Area under the function for 24-hour pulse pressure**

| **Variables** | **B** | **SE** | **p-value** | **Exp(B)** | **CI 95% (lower–upper)** | |
| --- | --- | --- | --- | --- | --- | --- |
| AUF 24-hPP (mmHg) | 0.002 | 0.001 | 0.015 | 1.0020 | 1.0001 | 1.0039 |
| Adherence (yes) | -0.776 | 0.385 | 0.044 | 0.460 | 0.216 | 0.979 |
| Statin use (yes) | 0.442 | 0.331 | 0.182 | 1.556 | 0.813 | 2.979 |
| FPG (mg/dL) | 0.016 | 0.010 | 0.089 | 1.016 | 0.998 | 1.036 |
| B_0_ | -2.855 | 1.033 | 0.006 | 0.058 |  | |

Logistic regression model elucidating the relationship between nSBP and Lp(a) levels are presented. The model incorporated data from 227 patients. ensuring no missing data. The variables to be controlled were adherence. statin use. physical activity. FPG. and WC. Omnibus test of model coefficients (p-value) = 0.004. Hosmer-Lemeshow test (p-value) = 0.233.

1. **Area under the function for daytime pulse pressure**

| **Variables** | **B** | **SE** | **p-value** | **Exp(B)** | **CI 95% (lower–upper)** | |
| --- | --- | --- | --- | --- | --- | --- |
| Adherence (yes) | -0.773 | 0.384 | 0.044 | 0.461 | 0.217 | 0.980 |
| Statin use (yes) | 0.497 | 0.329 | 0.130 | 1.644 | 0.863 | 3.132 |
| FPG (mg/dL) | 0.017 | 0.010 | 0.072 | 1.017 | 0.998 | 1.036 |
| AUF dPP | 0.002 | 0.001 | 0.045 | 1.0020 | 1.0001 | 1.0042 |
| B_0_ | -3.954 | 1.226 | 0.001 | 0.019 |  |  |

Logistic regression model elucidating the relationship between AUF dPP and Lp(a) levels are presented. The model incorporated data from 227 patients. ensuring no missing data. The variables to be controlled were adherence. statin use. physical activity. FPG. and WC. Omnibus tests of model coefficients (p-value) = 0.011. Hosmer-Lemeshow test (p-value) = 0.486.

1. **Area under the function for night-time pulse pressure**

| **Variables** | **B** | **SE** | **p-value** | **Exp(B)** | **CI 95% (lower–upper)** | |
| --- | --- | --- | --- | --- | --- | --- |
| Adherence (yes) | -0.637 | 0.372 | 0.087 | 0.529 | 0.255 | 1.096 |
| FPG (mg/dL) | 0.018 | 0.010 | 0.061 | 1.018 | 0.999 | 1.037 |
| AUF nPP (mmHg) | 0.005 | 0.002 | 0.019 | 1.0046 | 1.0010 | 1.0085 |
| B_0_ | -4.135 | 1.212 | 0.001 | 0.016 |  |  |

Logistic regression model elucidating the relationship between AUF nPP and Lp(a) levels are presented. The model incorporated data from 227 patients. ensuring no missing data. The variables to be controlled were adherence. statin use. physical activity. FPG. and WC. Omnibus test of model coefficients (p-value)= 0.007. Hosmer-Lemeshow test (p-value)= 0.931.

**Abbreviations.** Lp(a)–Lipoprotein(a); FPG–Fasting plasma glucose; SBP―Systolic blood pressure; 24-hSBP―Average 24-h SBP; dSBP―Average daytime SBP; nSBP―Average night-time SBP; DBP―Diastolic blood pressure; 24-hDBP―Average 24-h DBP; dDBP―Average daytime DBP; nDBP―Average night-time DBP; PP― Pulse pressure; 24-hPP― Average 24-h PP; dPP― Average daytime PP; nPP― Average night-time PP; AUF―Area under the function (calculation as the integral of the function by Simpson's method); AUF_24-hSBP―AUF of 24-hSBP; AUF_dSBP― AUF of daytime SBP; AUF_nSBP AUF of night-time SBP; DBP―Diastolic blood pressure; AUF_24-hDBP― AUF of 24-h DBP; AUF_dDBP― AUF of daytime DBP; AUF_nDBP― AUF of night-time DBP; AUF_24-hPP― AUF of 24-h Pulse pressure; AUF_dPP― AUF of daytime PP; AUF_nPP― AUF of Night-time PP; mmHg―Millimeter of mercury; %―Percentage; nmol―Nanomol; l―Liter.
