# Supplementary material for: Assessing the relationship between lipoprotein(a) levels and blood pressure among hypertensive patients beyond conventional measures. An observational study
Source: Sci Rep. 2024 Jun 23;14:14433. doi: 10.1038/s41598-024-65231-w (PMC11194270; doi:10.1038/s41598-024-65231-w)
Supplement: Supplementary file 1 — Supplementary Information. [file 41598_2024_65231_MOESM1_ESM.zip › Supplementary Appendix 1.docx]

**MATERIALS AND METHODS (Supplementary data)**

**Inclusion and exclusion criteria**

The presence of symptoms or signs suggestive of heart failure (HF) or the confirmation of HF diagnosis through transthoracic echocardiography, were exclusion criteria. Patients with a strong clinical suspicion of chronic obstructive pulmonary disease or a confirmed diagnosis based on spirometry results were also ineligible. Renal function was assessed in accordance with the recommendations provided by the Chronic Kidney Disease (CKD) guidelines, and patients with a reduced estimated glomerular filtration rate or persistent albuminuria were excluded. Individuals engaged in elite sports or undertaking high/very high-intensity physical activity were likewise excluded from the study [19].

**Parameters of 24-hour ABPM collection**

Blood pressure variability was considered according to the standard deviation (SD) values of 24-hour ABPM recordings. Blood pressure load was defined for each index as the percentage ratio between (a) the number of BP measurements equal to or above the threshold (24-h SBP: 130 mmHg, dSBP: 135 mmHg, nSBP: 120 mmHg, 24-h DBP: 80 mmHg, dDBP: 85 mmHg, nDBP: 70 mmHg) and (b) the total number of BP measurements for a period of time, thus computing the BP load variables. Pulse pressure load was defined as the percentage ratio between (a) the number of PP measurements equal to or above the threshold (24-h PP, dPP and nPP: 60 mmHg) and (b) the total number of PP measurements for a period of time, thus computing the PP load variables [7, 8].

**Laboratory variables**

Total cholesterol, HDL-c and TG concentrations were determined via turbidimetry using an Advia 2400 Chemistry System (Siemens Heathcare Diagnostic, Tarrytown, USA). Low-density lipoprotein cholesterol levels were computed using the Friedewald equation, except when TG levels exceeded 400 mg/dL, in which case LDL-c was directly measured by turbidimetry using an Advia 2400 Chemistry System (Siemens Heathcare Diagnostic, Tarrytown, USA). All measurements were expressed in mg/dL. Lipoprotein (a) levels were assessed through turbidimetry using a Binding Site Optilite system (The Binding Site Group Ltd., Birmingham, UK), with measurements recorded in nmol/L.

**RESULTS (Supplementary data)**

**Descriptive and univariate analysis**

*Laboratory variables*

In terms of glycemic parameters, the median FPG and glycated hemoglobin (HbA1c) levels fell within the physiological range. Nevertheless, we noted slightly higher FPG levels in patients with Lp(a) levels exceeding the 75th percentile (125 nmol/L); although both groups maintained FPG levels within the normoglycemic range. Furthermore, we observed no significant differences in HbA1c levels between the compared groups. As for the lipid profile, the levels of lipoproteins (TG, TC, LDL-c and HDL-c) remained within the reference range and exhibited no differences between the compared groups (refer to Table 1).

*Blood pressure indices (blood pressure variability)*

When examining BP variability, we found that the standard deviation values for each BP index remained at or below 15 mmHg for SBP and 10 mmHg for DBP indices across all groups. When analyzing the data by groups, we found a trend towards increased PP variability among patients exhibiting higher Lp(a) levels with results reaching statistical significance for nPP (refer to Supplementary Table 1(b) and Figure 1).
