# Supplementary figures and images for: Assessing the relationship between lipoprotein(a) levels and blood pressure among hypertensive patients beyond conventional measures. An observational study
Source: Sci Rep. 2024 Jun 23;14:14433. doi: 10.1038/s41598-024-65231-w (PMC11194270; doi:10.1038/s41598-024-65231-w)

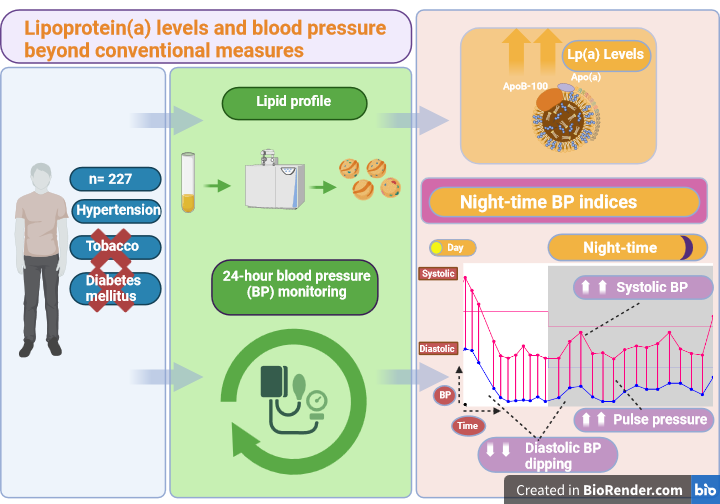

Supplement: Supplementary file 1 — Supplementary Information. [file 41598_2024_65231_MOESM1_ESM.zip › Graphical abstract.png]

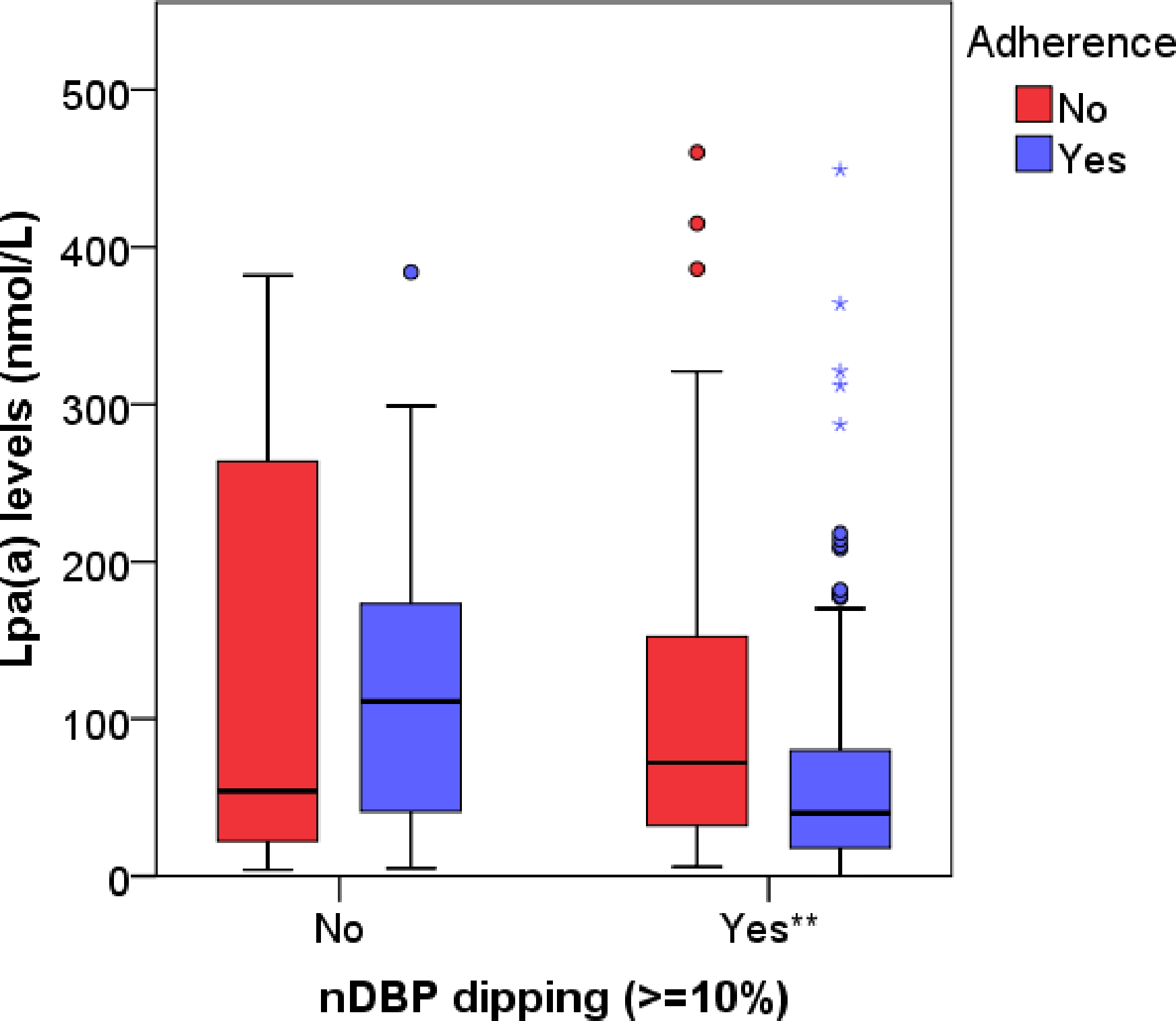

Supplement: Supplementary file 1 — Supplementary Information. [file 41598_2024_65231_MOESM1_ESM.zip › Supplementary Figure 1.tif]
