# Supplementary material for: Assessing the relationship between lipoprotein(a) levels and blood pressure among hypertensive patients beyond conventional measures. An observational study
Source: Sci Rep. 2024 Jun 23;14:14433. doi: 10.1038/s41598-024-65231-w (PMC11194270; doi:10.1038/s41598-024-65231-w)
Supplement: Supplementary file 1 — Supplementary Information. [file 41598_2024_65231_MOESM1_ESM.zip › Supplementary Tables 2.doc]

***Supplementary Tables 2. Linear correlation analysis.***

(a) **Average Blood pressure índices.**

| **Spearman correlations** | | | | | | | | | | | |
| --- | --- | --- | --- | --- | --- | --- | --- | --- | --- | --- | --- |
|  | | | Lp(a) | 24-hSBP | dSBP | nSBP | 24-hPP | dPP | nPP | nSBP dipping | nDBP dipping |
| Rho de Spearman | Lp(a) (nmol/L) | Coefficient (Rho) | 1.000 |  |  |  |  |  |  |  |  |
|  |  | Sig. (two-tailed) | . |  |  |  |  |  |  |  |  |
|  |  | N | 227 |  |  |  |  |  |  |  |  |
|  | 24-hSBP (mmHg) | Coefficient (Rho) | 0.107 | 1.000 |  |  |  |  |  |  |  |
|  |  | Sig. (two-tailed) | 0.108 | . |  |  |  |  |  |  |  |
|  |  | N | 227 | 227 |  |  |  |  |  |  |  |
|  | dSBP (mmHg) | Coefficient (Rho) | 0.087 | 0.977^**^ | 1.000 |  |  |  |  |  |  |
|  |  | Sig. (two-tailed) | 0.189 | <0.001 | . |  |  |  |  |  |  |
|  |  | N | 227 | 227 | 227 |  |  |  |  |  |  |
|  | nSBP (mmHg) | Coefficient (Rho) | 0.141^*^ | 0.865^**^ | 0.761^**^ | 1.000 |  |  |  |  |  |
|  |  | Sig. (two-tailed) | 0.034 | <0.001 | <0.001 | . |  |  |  |  |  |
|  |  | N | 227 | 227 | 227 | 227 |  |  |  |  |  |
|  | 24-hPP (mmHg) | Coefficient (Rho) | 0.135^*^ | 0.700^**^ | 0.683^**^ | 0.650^**^ | 1.000 |  |  |  |  |
|  |  | Sig. (two-tailed) | 0.043 | <0.001 | <0.001 | <0.001 | . |  |  |  |  |
|  |  | N | 227 | 227 | 227 | 227 | 227 |  |  |  |  |
|  | dPP (mmHg) | Coefficient (Rho) | 0.136^*^ | 0.680^**^ | 0.683^**^ | 0.587^**^ | 0.987^**^ | 1.000 |  |  |  |
|  |  | Sig. (two-tailed) | 0.041 | <0.001 | <0.001 | <0.001 | <0.001 | . |  |  |  |
|  |  | N | 227 | 227 | 227 | 227 | 227 | 227 |  |  |  |
|  | nPP (mmHg) | Coefficient (Rho) | 0.123 | 0.649^**^ | 0.582^**^ | 0.750^**^ | 0.898^**^ | 0.829^**^ | 1.000 |  |  |
|  |  | Sig. (two-tailed) | 0.064 | <0.001 | <0.001 | <0.001 | <0.001 | <0.001 | . |  |  |
|  |  | N | 227 | 227 | 227 | 227 | 227 | 227 | 227 |  |  |
|  | nSBP dipping (%) | Coefficient (Rho) | -0.088 | -0.012 | 0.161^*^ | -0.474^**^ | -0.070 | 0.019 | -0.332^**^ | 1.000 |  |
|  |  | Sig. (two-tailed) | 0.188 | 0.860 | 0.015 | <0.001 | 0.292 | 0.781 | <0.001 | . |  |
|  |  | N | 227 | 227 | 227 | 227 | 227 | 227 | 227 | 227 |  |
|  | nDBP dipping (%) | Coefficient (Rho) | -0.163^*^ | -0.068 | 0.074 | -0.447^**^ | -0.165^*^ | -0.139^*^ | -0.251^**^ | 0.832^**^ | 1.000 |
|  |  | Sig. (two-tailed) | 0.014 | 0.308 | 0.268 | <0.001 | 0.013 | 0.036 | <0.001 | <0.001 | . |
|  |  | N | 227 | 227 | 227 | 227 | 227 | 227 | 227 | 227 | 227 |

*. P< 0.05

**. P< 0.01

(b) **Blood pressure load.**

| **Spearman correlations** | | | | | | | | | |
| --- | --- | --- | --- | --- | --- | --- | --- | --- | --- |
|  | | | Lp(a) | 24-hSBP load | dSBP load | nSBP load | 24-hPP load | dPP load | nPP load |
|  | Lp(a) (nmol/L) | Coefficient (Rho) | 1.000 |  |  |  |  |  |  |
|  |  | Sig. (two-tailed) | . |  |  |  |  |  |  |
|  |  | N | 227 |  |  |  |  |  |  |
|  | 24-hSBP load (%) | Coefficient (Rho) | 0.106 | 1.000 |  |  |  |  |  |
|  |  | Sig. (two-tailed) | 0.110 | . |  |  |  |  |  |
|  |  | N | 227 | 227 |  |  |  |  |  |
|  | dSBP load (%) | Coefficient (Rho) | 0.099 | 0.973^**^ | 1.000 |  |  |  |  |
|  |  | Sig. (two-tailed) | 0.139 | < 0.001 | . |  |  |  |  |
|  |  | N | 227 | 227 | 227 |  |  |  |  |
|  | nSBP load (%) | Coefficient (Rho) | 0.112 | 0.846^**^ | 0.717^**^ | 1.000 |  |  |  |
|  |  | Sig. (two-tailed) | 0.092 | < 0.001 | < 0.001 | . |  |  |  |
|  |  | N | 227 | 227 | 227 | 227 |  |  |  |
|  | 24-hPP load (%) | Coefficient (Rho) | 0.130 | 0.703^**^ | 0.685^**^ | 0.618^**^ | 1.000 |  |  |
|  |  | Sig. (two-tailed) | 0.051 | < 0.001 | < 0.001 | < 0.001 | . |  |  |
|  |  | N | 227 | 227 | 227 | 227 | 227 |  |  |
|  | dPP load (%) | Coefficient (Rho) | 0.115 | 0.680^**^ | 0.680^**^ | 0.553^**^ | 0.983^**^ | 1.000 |  |
|  |  | Sig. (two-tailed) | 0.084 | < 0.001 | < 0.001 | < 0.001 | < 0.001 | . |  |
|  |  | N | 227 | 227 | 227 | 227 | 227 | 227 |  |
|  | nPP load (%) | Coefficient (Rho) | 0.164^*^ | 0.560^**^ | 0.482^**^ | 0.658^**^ | 0.770^**^ | 0.667^**^ | 1.000 |
|  |  | Sig. (two-tailed) | 0.013 | < 0.001 | < 0.001 | < 0.001 | < 0.001 | < 0.001 | . |
|  |  | N | 227 | 227 | 227 | 227 | 227 | 227 | 227 |

*. P< 0.05

**. P< 0.01

(c) **Blood pressure variability.**

| **Spearman correlations** | | | | |
| --- | --- | --- | --- | --- |
|  | | | Lp(a) | nPP SD |
|  | Lp(a) (nmol/L) | Coefficient (Rho) | 1.000 |  |
|  |  | Sig. (two-tailed) | . |  |
|  |  | N | 227 |  |
|  | nPP SD (mmHg) | Coefficient (Rho) | 0.115 | 1.000 |
|  |  | Sig. (two-tailed) | 0.083 | . |
|  |  | N | 227 | 227 |

*. P< 0.05

**. P< 0.01

(d) **Area under the function over time of blood pressure levels.**

| **Spearman correlations** | | | | | | | | | |
| --- | --- | --- | --- | --- | --- | --- | --- | --- | --- |
|  | | | Lp(a) | AUF 24-hSBP | AUF nSBP | AUF nDBP dipping | AUF 24-hPP | AUF dPP | AUF nPP |
| Rho de Spearman | Lp(a) (nmol/L) | Coeffcient (Rho) | 1.000 |  |  |  |  |  |  |
|  |  | Sig. (two-tailed) | . |  |  |  |  |  |  |
|  |  | N | 227 |  |  |  |  |  |  |
|  | AUF 24-hSBP (mmHg) | Coeffcient (Rho) | 0.102 | 1.000 |  |  |  |  |  |
|  |  | Sig. (two-tailed) | 0.125 | . |  |  |  |  |  |
|  |  | N | 227 | 227 |  |  |  |  |  |
|  | AUF nSBP (mmHg) | Coeffcient (Rho) | 0.140^*^ | 0.470^**^ | 1.000 |  |  |  |  |
|  |  | Sig. (two-tailed) | 0.035 | <0.001 | . |  |  |  |  |
|  |  | N | 227 | 227 | 227 |  |  |  |  |
|  | AUF nDBP dipping (%) | Coeffcient (Rho) | -0.146^*^ | 0.114 | -0.755^**^ | 1.000 |  |  |  |
|  |  | Sig. (two-tailed) | 0.028 | 0.088 | <0.001 | . |  |  |  |
|  |  | N | 227 | 227 | 227 | 227 |  |  |  |
|  | AUF 24-hPP (mmHg) | Coeffcient (Rho) | 0.128 | 0.693^**^ | 0.358^**^ | 0.018 | 1.000 |  |  |
|  |  | Sig. (two-tailed) | 0.054 | <0.001 | <0.001 | 0.789 | . |  |  |
|  |  | N | 227 | 227 | 227 | 227 | 227 |  |  |
|  | AUF dPP (mmHg) | Coeffcient (Rho) | 0.080 | 0.651^**^ | 0.051 | 0.324^**^ | 0.901^**^ | 1.000 |  |
|  |  | Sig. (two-tailed) | 0.230 | <0.001 | 0.447 | <0.001 | <0.001 | . |  |
|  |  | N | 227 | 227 | 227 | 227 | 227 | 227 |  |
|  | AUF nPP (mmHg) | Coeffcient (Rho) | 0.151^*^ | 0.456^**^ | 0.826^**^ | -0.582^**^ | 0.669^**^ | 0.356^**^ | 1.000 |
|  |  | Sig. (two-tailed) | 0.023 | <0.001 | <0.001 | <0.001 | <0.001 | <0.001 | . |
|  |  | N | 227 | 227 | 227 | 227 | 227 | 227 | 227 |

*. P< 0.05

**. P< 0.01

**Abbreviations.** Lp(a)–Lipoprotein(a); 24-hSBP―Average 24-h SBP; dSBP―Average daytime SBP; nSBP―Average night-time SBP; DBP―Diastolic blood pressure; 24-hDBP―Average 24-h DBP; dDBP―Average daytime DBP; nDBP―Average night-time DBP; PP― Pulse pressure; 24-hPP― Average 24-h PP; dPP― Average daytime PP; nPP― Average night-time PP; AUF―Area under the function (calculation as the integral of the function by Simpson's method); AUF_24-hSBP―AUF of 24-hSBP; AUF_dSBP― AUF of daytime SBP; AUF_nSBP AUF of night-time SBP; DBP―Diastolic blood pressure; AUF_24-hDBP― AUF of 24-h DBP; AUF_dDBP― AUF of daytime DBP; AUF_nDBP― AUF of night-time DBP; AUF_24-hPP― AUF of 24-h Pulse pressure; AUF_dPP― AUF of daytime PP; AUF_nPP― AUF of Night-time PP; mmHg―Millimeter of mercury; %―Percentage; nmol―Nanomol; l―Liter.
